# Supplementary material for: 2D MXene Nanosheets with ROS Scavenging Ability Effectively Delay Osteoarthritis Progression
Source: Nanomaterials (Basel). 2024 Sep 29;14(19):1572. doi: 10.3390/nano14191572 (PMC11478061; doi:10.3390/nano14191572)
Supplement: Supplementary file 1 [file nanomaterials-14-01572-s001.zip › nanomaterials-3131823-supplementary.pdf]

*Supplementary Materials*

# 2D MXene Nanosheets with ROS Scavenging Ability Effectively Delay Osteoarthritis Progression

**Hongqi Zhao [1,†], Tianqi Wang [1,†], Xuan Fang [1], Tao Xu [1], Jian Li [1,2], Shaoze Jing [1,2], Guangzi Chen [1], Yang Liu [1,*] and Gaohong Sheng [1,*]**

1 Department of Orthopedics, Tongji Hospital, Tongji Medical College, Huazhong University of Science and Technology, Wuhan 430030, China.
2 Third Hospital of Shanxi Medical University, Shanxi Bethune Hospital, Shanxi Academy of Medical Sciences Tongji Shanxi Hospital, Taiyuan 030032, China.
* Correspondence: yangliu@tjh.tjmu.edu.cn (Y.L.); gaohongsheng@hust.edu.cn (G.S.)
† These authors contributed equally to this work.

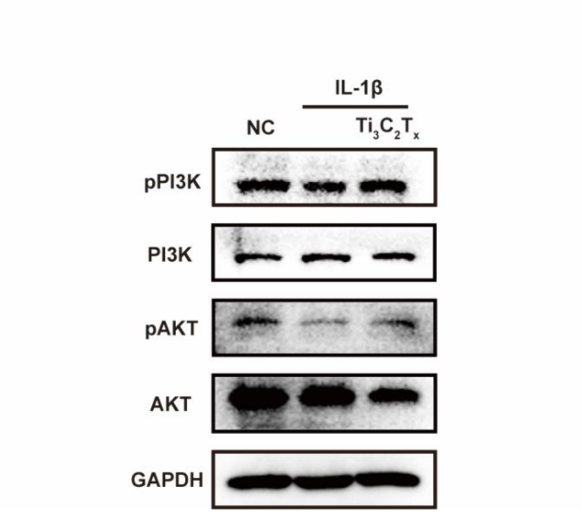


**Figure S1.** the western blotting results of PI3K/Akt pathway by $Ti_3C_2T_x$ nanosheets.

**Table S1.** The primer sequences used in RT-qPCR analysis.

| Gene name | | Primer sequence |
|---|---|---|
| Col2 | Forward | ACGCTCAAGTCGCTGAACAAC |
| | Reverse | AATCCAGTAGTCTCCGCTCTTCC |
| MMP13 | Forward | CAAGATGTGGAGTGCCTGATGTG |
| | Reverse | TTAAGTTTGTTTGGGACCATTTGAGTG |
| ACAN | Forward | CAGAACCTTCGCTCCAATGAC |
| | Reverse | CCTCAATGCCATGCATCACTT |
| iNOS | Forward | TCTTGGAGCGAGTTGTGGATTGTTC |
| | Reverse | AGTGATGTCCAGGAAGTAGGTGAGG |
| COX2 | Forward | TGACAGCCCACCAACTTACAATG |
| | Reverse | TCATCAGCCACAGGAGGAAGG |
| ACTB | Forward | TGTCACCAACTGGGACGATA |
| | Reverse | GGGGTGTTGAAGGTCTCAAA |
